# Dynamic GLUT4 sorting through a syntaxin-6 compartment in muscle cells is derailed by insulin resistance-causing ceramide

**DOI:** 10.1242/bio.20147898

**Published:** 2014-04-04

**Authors:** Kevin P. Foley, Amira Klip

**Affiliations:** 1 Program in Cell Biology, The Hospital for Sick Children, Toronto, ON M5G 1X8, Canada; 2 Department of Biochemistry, University of Toronto, Toronto, ON M5S 1A8, Canada

**Keywords:** GLUT4, Syntaxin-6, Insulin-responsiveness, Intracellular sorting, Transferrin recycling

## Abstract

GLUT4 constitutively recycles between the plasma membrane and intracellular depots. Insulin shifts this dynamic equilibrium towards the plasma membrane by recruiting GLUT4 to the plasma membrane from insulin-responsive vesicles. Muscle is the primary site for dietary glucose deposition; however, how GLUT4 sorts into insulin-responsive vesicles, and if and how insulin resistance affects this process, is unknown. In L6 myoblasts stably expressing myc-tagged GLUT4, we analyzed the intracellular itinerary of GLUT4 as it internalizes from the cell surface and examined if such sorting is perturbed by C2-ceramide, a lipid metabolite causing insulin resistance. Surface-labeled GLUT4*myc* that internalized for 30 min accumulated in a Syntaxin-6 (Stx6)- and Stx16-positive perinuclear sub-compartment devoid of furin or internalized transferrin, and displayed insulin-responsive re-exocytosis. C2-ceramide dispersed the Stx6-positive sub-compartment and prevented insulin-responsive re-exocytosis of internalized GLUT4*myc*, even under conditions not affecting insulin-stimulated signaling towards Akt. Microtubule disruption with nocodazole prevented pre-internalized GLUT4*myc* from reaching the Stx6-positive perinuclear sub-compartment and from undergoing insulin-responsive exocytosis. Removing nocodazole allowed both parameters to recover, suggesting that the Stx6-positive perinuclear sub-compartment was required for GLUT4 insulin-responsiveness. Accordingly, Stx6 knockdown inhibited by ∼50% the ability of internalized GLUT4*myc* to undergo insulin-responsive re-exocytosis without altering its overall perinuclear accumulation. We propose that Stx6 defines the insulin-responsive compartment in muscle cells. Our data are consistent with a model where ceramide could cause insulin resistance by altering intracellular GLUT4 sorting.

## INTRODUCTION

The regulation of glucose transport into muscle and fat cells is essential to glucose homeostasis, and is mediated by the Glucose Transporter 4 (GLUT4) protein. In resting cells GLUT4 constitutively recycles between the plasma membrane and intracellular depots in a dynamic equilibrium that favors its intracellular localization (Foley et al., 2011). To increase glucose uptake into muscle and adipose tissue, insulin signals promote translocation of GLUT4 to the plasma membrane, but, surprisingly, the intracellular localization of the GLUT4-retaining, insulin-responding intracellular compartment (commonly termed GLUT4-storage vesicles or insulin-responding vesicles, respectively GSV or IRV) remains unknown (Foley et al., 2011; Rowland et al., 2011). Instead, this compartment is defined functionally (Govers et al., 2004; Karylowski et al., 2004) and can be crudely recovered by subcellular fractionation (Kupriyanova et al., 2002), i.e. GSV/IRV are terms given to subcellular fractions containing insulin-responsive GLUT4.

Obesity and Type 2 Diabetes are characterized by insulin resistance. A prevailing hypothesis is that the accumulation of lipids or lipid by-products (diacylglycerol, ceramides) in muscle and adipose tissues can cause inflammation and insulin resistance (reviewed by Chavez and Summers, 2012; Samuel et al., 2010; Shoelson et al., 2007). Defective GLUT4 translocation in muscle is a key feature of insulin resistance (Klip et al., 1990; Ryder et al., 2000; Zierath et al., 1996), but to date, defects are assigned to alterations in insulin-derived signals and the possible contribution of proper intracellular sorting of GLUT4 has not been analyzed. Cellular studies have revealed that a cell permeable ceramide analog, C2-ceramide (C2-cer), inhibits Akt activation and GLUT4 translocation in response to insulin without affecting upstream IRS-1 or PI3K activation (Hajduch et al., 2001; JeBailey et al., 2007; Summers et al., 1998; Wang et al., 1998). However, difficulty in defining the intracellular localization of the GSV/IRV has left untested the possibility that defective GLUT4 sorting may also contribute to C2-ceramide induced insulin resistance.

Here we explore how GLUT4 sorts from the plasma membrane of L6 muscle cells into compartments that define acquisition of insulin responsiveness, and examine if C2-ceramide, which confers insulin resistance, can affect GLUT4 sorting into these compartments. Earlier studies in 3T3-L1 adipocytes had highlighted the participation of Syntaxin 6 (Stx6) in GLUT4 traffic (Perera et al., 2003; Shewan et al., 2003) (see Discussion). Our results further build upon this knowledge, focusing on GLUT4 traffic in muscle cells. By tracking the intracellular sorting of GLUT4 after pulse-labeling at the cell surface, we show that GLUT4 sorts into a perinuclear sub-compartment that is positive for Stx6 and devoid of furin or transferrin receptor (TfR); moreover, we show that C2-ceramide prevents this sorting in parallel with inhibiting GLUT4 re-exocytosis, even when signaling to Akt is allowed to resume. GLUT4 arrival at the Stx6-positive perinuclear sub-compartment correlates with GLUT4 acquisition of insulin-responsive exocytosis and Stx6 knockdown inhibits this re-exocytosis. We further show that insulin resistance can arise from defective GLUT4 sorting, and that ceramide disperses the GLUT4 and Stx6-positive compartment. Our data are consistent with a model where ceramide would cause insulin resistance by altering intracellular GLUT4 sorting.

## MATERIALS AND METHODS

### Antibodies, reagents, and siRNA

Rabbit polyclonal anti-*myc* antibody, mouse monoclonal anti-actinin-1 antibody, and DMSO were from Sigma–Aldrich (St Louis, MO, USA). Mouse monoclonal anti-Stx6 antibody was from BD Transduction Laboratories (San Jose, CA, USA). Rabbit polyclonal anti-Stx6 and anti-Stx16 antibodies were from Synaptic Systems (Goettingen, Germany). Mouse monoclonal anti-Tubulin antibody was from Abcam (Cambridge, MA, USA). Human holo-transferrin conjugated to A488 was from Invitrogen (Grand Island, NY, USA). Mouse anti-*myc* (c-*Myc* 9E10) and rabbit anti-furin (H-220) were from Santa Cruz Biotechnology (Dallas, TX, USA). Polyclonal anti-P-Akt(308) and P-Akt(473) were obtained from Cell Signaling Technology (Danvers, MA, USA). Cy3- and A488-conjugated donkey anti-rabbit and donkey anti-mouse secondary antibodies and horseradish peroxidase (HRP)-conjugated goat anti-rabbit secondary antibodies were purchased from Jackson ImmunoResearch Laboratories (West Grove, PA, USA). Nocodazole was purchased from EMD Biosciences Inc. (Darmstadt, Germany) (10 mM stock in DMSO) and C2-ceramide was purchased from Enzo Life Sciences (Farmingdale, NY, USA) (50 mM stock in DMSO). Pre-designed siRNA for Stx6 (siStx6: 5′-CCGAGTCATCAGAAGAACTAA-3′) and non-related (siNR: 5′-AATAAGGCTATGAAGAGATA C-3′) were from Qiagen (Valencia, CA, USA). Human insulin was purchased from Eli Lilly (Indianapolis, IN, USA).

### Cell culture and transfections

The rat L6 muscle cell line stably expressing GLUT4 with an exofacial *myc* epitope tag (L6GLUT4*myc*) was cultured as described previously (Ueyama et al., 1999). Transfection of siRNA was performed using jetPRIME reagent according to the manufacturer's protocol (Polyplus transfection, Illkirch, France). Cells were transfected with 200 nM siRNA for 24 h and then cultured for 48 h. For insulin-responsive GLUT4*myc* re-exocytosis experiments, cells were grown in 24-well plates to confluence. For immunofluorescence experiments, cells were re-seeded onto glass coverslips 24–48 h before experiments. For nocodazole and C2-ceramide experiments cells were grown to confluence in 24-well plates (insulin-responsive GLUT4*myc* re-exocytosis) or seeded onto coverslips 24 h before use (immunofluorescence).

### Imaging GLUT4 internalization in single cells

The GLUT4*myc* internalization protocol was adapted from previously established protocols (Ishikura et al., 2010). L6GLUT4*myc* cells were serum starved for 2 h before being washed twice in PBS+ and placed in blocking buffer (5% goat serum in PBS+) for 20 min on ice. Cell surface GLUT4*myc* was pulse-labeled with rabbit anti-*myc* antibody (1:250) at 4°C for 1 h before cells were washed 5× in PBS+ and re-warmed in serum free medium at 37°C for indicated times. Cells were then fixed and permeabilized for detection of internalized GLUT4*myc* by secondary antibody conjugated to fluorophore (1:400). Endogenous Stx6 was detected by mouse anti-Stx6 antibody (1:100) and fluorophore conjugated secondary antibody (1:500) after permeabilization.

For Tfn-A488 experiments, Tfn-A488 (50 µg/mL) in serum free medium supplemented with 1% bovine serum albumin (BSA) was added to cells for 30 min prior to cell surface GLUT4*myc* detection. Tfn-A488 was kept present during cell re-warm after surface GLUT4*myc* labeling. Cells were fixed for 1 h in 4% PFA at room temperature.

For nocodazole experiments, 3 µM nocodazole was added during the 30 min cell re-warm after surface GLUT4*myc* pulse-labeling. During nocodazole recovery, cells were washed once with PBS and placed in serum free medium for 5, 10, or 15 min after 25 min nocodazole treatment during cell re-warm. For C2-ceramide treatment, 50 µM C2-ceramide was added during the initial 2 h serum starvation prior to the pulse-labeling of cell surface GLUT4*myc* and remained present during the 30 min re-warm. During C2-ceramide recovery, cells were washed once with PBS and placed in serum free medium for 15 min after the 2 h C2-ceramide treatment during serum starvation. Cell surface GLUT4*myc* was then pulse-labeled and cells were re-warmed for 30 min in the absence of C2-ceramide (total 45 min recovery).

### Insulin-responsive GLUT4 re-exocytosis

Cells were serum starved for 2 h prior to 15 min stimulation with 100 nM insulin. Cell surface GLUT4*myc* was pulse-labeled at 4°C with anti-*myc* antibody. Cells were then washed and re-warmed to 37°C in serum free medium for indicated times (in most assays 30 min) and treated with or without insulin for 5 or 10 min, to stimulate GLUT4*myc* re-exocytosis. Cells were placed on ice, fixed, and surface GLUT4*myc* was detected by adding secondary anti-rabbit antibody conjugated to horseradish peroxidase as previously described (Ishikura et al., 2010). Where indicated, nocodazole and C2-ceramide treatments were performed as described above. For these re-exocytosis experiments, nocodazole was never present during the insulin-stimulated re-exocytosis step. Nocodazole recovery (5, 10, or 15 min) included time of insulin-stimulated re-exocytosis. Where used, C2-ceramide was present during the entire re-exocytosis protocol. The time of insulin-stimulated re-exocytosis was in addition to C2-ceramide recovery (total 45 min recovery then 10 min insulin).

### GLUT4 endocytosis

The disappearance of GLUT4*myc* from the plasma membrane was measured as previously described (Antonescu et al., 2008). Briefly, cell surface GLUT4*myc* was pulse-labeled at 4°C. Cells were washed and re-warmed to 37°C for 10 min to induce GLUT4 endocytosis. Cells were fixed on ice and GLUT4*myc* remaining at the cell surface was detected by horseradish peroxidase-conjugated secondary antibody.

### Detection of total GLUT4*myc* by immunofluorescence

Detection of total GLUT4*myc* in single cells was performed as previously described (Ishikura et al., 2010). Briefly, L6GLUT4*myc* cells were serum starved 2 h before being fixed in 3% PFA, permeabilized with 0.1% Triton for 15 min, placed in blocking buffer for 20 min, and labeled with primary antibodies for 1 h at RT. GLUT4*myc* was labeled with mouse anti-*myc* (9E10) antibody (1:100). Endogenous proteins were labeled as follows: rabbit anti-Stx6 (1:750), rabbit anti-Stx16 (1:750), and rabbit anti-furin (1:500). Recycling TfnR was labeled with Tfn-A488 conjugates. Cells were incubated with Tfn-A488 for 30 min prior to fixation (50 µg/mL in serum free medium supplemented with 1% BSA). Fixation for 1 h at RT with 4% PFA was required for Tfn labeling. Appropriate fluorophore conjugated secondary antibodies were then used to label GLUT4*myc* and markers for 1 h at RT before coverslips were mounted for imaging.

### Detection of cell surface GLUT4*myc*

Cell surface GLUT4*myc* was detected as described previously, with slight modifications (Ishikura et al., 2010). Cells were serum starved for 2 h prior to 15 min stimulation with 100 nM insulin. Cells were then fixed in 3% PFA for 30 min (10 min on ice and 20 min at RT), washed with 0.1 M glycine for 10 min, and blocked in 5% goat serum for 20 min. Cell surface GLUT4*myc* was labeled at RT (rabbit anti-*myc* 1:500) and surface GLUT4*myc* detected by horse-radish peroxidase conjugated secondary antibody (1 h at RT, 1:1000) as previously described (Ishikura et al., 2010).

### Immunoblotting

To assess Stx6 knockdown, replicate wells from each experiment were lysed with 1% NP-40. Protein samples were resolved by 10% SDS-PAGE, transferred and immunoblotted with rabbit anti-Stx6 antibody (1:750). Actinin-1 was used loading control (1:10,000). To detect Akt phosphorylation, cells grown to confluence in 12-well plates were subjected to experimental treatments and then lysed and subjected to SDS-PAGE and blotting using anti-P-Akt-S473 (1:1000) and anti-P-Akt-T308 (1:1000).

### Spinning disk confocal fluorescence microscopy and image analysis

Fluorescent images were acquired with an Olympus IX81 inverted fluorescence microscope equipped with a 60× objective (1.35 NA), Hamamatsu C9100-13 back-thinned EM-CCD camera, and Yokogawa CSU X1 spinning disk confocal scan head (with Spectral Aurora Borealis upgrade). Images were acquired of multiple z-slices (0.3 µm) and collapsed *xy* projections presented. Image analysis was performed using Perkin Elmer Volocity software. Single cells were selected and the Pearson's Correlation calculated for the whole cell using Volocity software.

### Statistical analysis

Statistical analyses were carried out using Prism 4.0 software (GraphPad Software, San Diego, CA). Groups were compared using one-way analysis of variance Newman–Keuls post hoc analysis or Student T-Test. p<0.05 was considered statistically significant.

## RESULTS

Current models drawn from studies in 3T3-L1 adipocytes suggest that the intracellular stores of GLUT4 at steady-state include the recycling endosomes, the Trans-Golgi Network (TGN), and the GSV/IRV, the latter two compartments serving to sequester GLUT4 from recycling back to the plasma membrane (Blot and McGraw, 2008; Brewer et al., 2011; Foley et al., 2011; Stöckli et al., 2011). Although insulin-responding GLUT4-containing vesicles have been imaged within 200 nm of the plasma membrane of adipocytes (Bai et al., 2007; Chen et al., 2012; Huang et al., 2007; Lizunov et al., 2005; Xiong et al., 2010) and muscle cells (Boguslavsky et al., 2012; Sun et al., 2014), it is unknown how or where GSV/IRV are constituted to segregate away from constitutive recycling, as at steady-state GLUT4 is visualized both in the perinuclear region and in cytosolic vesicles (Boguslavsky et al., 2012; Chen et al., 2012; Foley et al., 2011; Randhawa et al., 2008; Xiong et al., 2010).

Part of the difficulty in ascertaining the localization of GSV/IRV in adipose cells (whether 3T3-L1 preadipocytes or mature primary adipocytes) is their limited cytosolic space, and this challenge is also encountered in mature skeletal muscle fibers. In contrast, muscle cells in culture present a rather unobstructed cytosolic space. Nonetheless, in all those systems, GLUT4 is largely detected at the perinuclear region, and in muscle cells and 3T3-L1 adipocytes a fraction of GLUT4 can be clearly observed to disperse into the cytosol upon insulin treatment (Boguslavsky et al., 2012; Fujita et al., 2010; Randhawa et al., 2008). Because the TGN localizes to the perinuclear region, recycling endosomes are both perinuclear and cytosolic, and the location of GSV/IRV is unknown, it is unclear where insulin responsiveness is acquired. Based on pioneering work by Mastick's and McGraw's groups (Blot and McGraw, 2008; Brewer et al., 2011; Habtemichael et al., 2011; Karylowski et al., 2004; Xiong et al., 2010; Zeigerer et al., 2002), we reasoned that the steady-state distribution of GLUT4 alone does not allow one to establish where insulin responsiveness is conferred, and that this impasse might be overcome by complementing imaging the GLUT4 steady-state distributions with analysis of GLUT4 as it returns from the cell surface and is then stimulated to re-externalize.

### GLUT4 concentrates in a perinuclear sub-compartment containing Stx6 and Stx16, but not furin

**Fig. 1.**
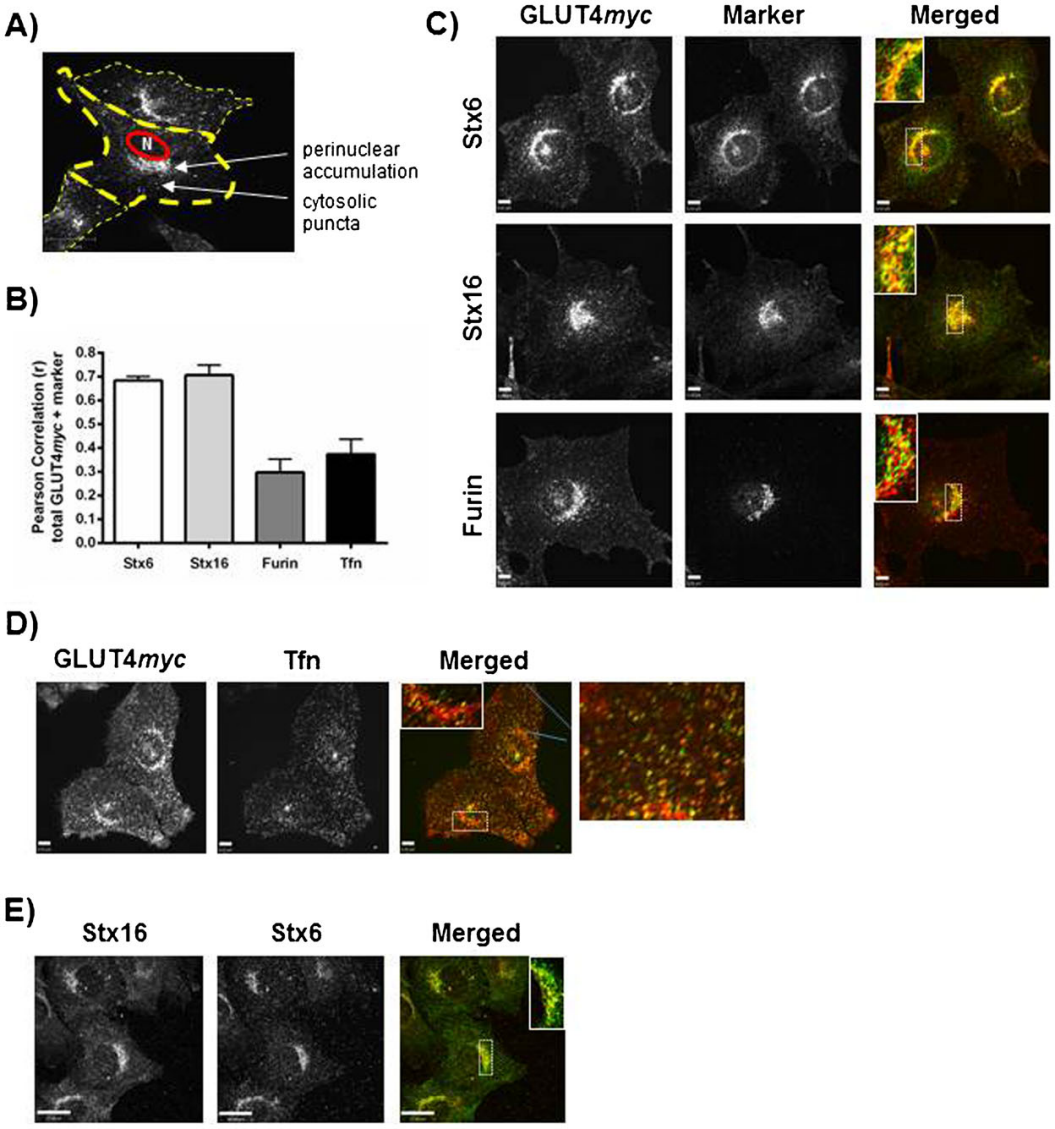
GLUT4 localizes to a Stx6- and Stx16-positive perinuclear sub-compartment that is devoid of recycling Tfn. (A) GLUT4*myc* localization in a resting L6 muscle cell. Cells were fixed, permeabilized, and labeled with mouse anti-*myc* antibody. (B) Quantification of GLUT4*myc* co-localization with markers of endomembrane compartments. Single cells were selected and Pearson correlations calculated using Volocity software. (Stx6, N = 4; Stx16, N = 3; Furin, N = 3; Tfn, N = 3). 8–15 cells were quantified in each experiment. (C) Cells were fixed, permeabilized, and labeled with mouse anti-*myc* antibody (red) and rabbit anti-Stx6, Stx16, or furin antibodies (green). Inset images show single perinuclear slices of the boxed regions. (D) Cells were incubated with Tfn conjugated to A488 fluorophore (green) for 30 min prior to being fixed, permeabilized, and labeled for GLUT4*myc* (red). Inset image shows single perinuclear slice of the boxed region. Far right column shows enlarged regions of collapsed image as outlined by blue lines. (E) Stx6 (red) and Stx16 (green) co-localize. Cells were treated as in panel C. Inset image shows single perinuclear slice of the boxed region. Scale bars: 6 µm (C,D), 17 µm (E).

In resting L6 muscle cells imaged by spinning disc confocal fluorescence microscopy, GLUT4*myc* at steady-state localizes to both cytosolic puncta and to the perinuclear region (Fig. 1A). The directionality of GLUT4 vesicle movement cannot be determined from studying steady-state GLUT4 localization, so cytosolic puncta may represent both retrograde and anterograde vesicles. Likewise, the population of perinuclear GLUT4 is not necessarily homogeneous and is maintained in dynamic equilibrium. Yet, defining the intracellular localization of GLUT4 is critical for understanding the regulation of GLUT4 dynamics.

We first characterized the localization of GLUT4*myc* in resting myocytes vis-à-vis endogenous markers of endomembrane compartments. As shown in Fig. 1, the perinuclear pool of GLUT4*myc* co-localizes with Syntaxins 6 and 16 (Stx6 and Stx16), but shows little overlap with the TGN-resident protein furin (Fig. 1C). Stx6 is a Q-SNARE involved in the retention of TGN resident molecules, retrograde traffic, and post-Golgi transport (Jung et al., 2012). It is also found in early endosomes where it functions in the endocytic recycling of defined cargo proteins (Jung et al., 2012). In L6 myoblasts, Stx6 and Stx16 also overlap considerably with each other (Fig. 1E). In the cytosol, GLUT4*myc* showed partial overlap with internalized Tfn in cytosolic puncta, indicative of GLUT4 presence in cytosolic early/recycling endosomes. However, the perinuclear sub-compartment populated by GLUT4*myc* was notably devoid of internalized Tfn (Fig. 1D). Pearson's correlations were used to quantify GLUT4*myc* co-localization with each of these protein markers (Fig. 1B). The Pearson's correlation coefficients reveal a strong correlation for GLUT4*myc* co-localization with Stx6 and Stx16 (0.68 and 0.70, respectively), which were comparable to the coefficient of co-localization of Stx6 with Stx16 (0.58, N = 2; 7 cells quantified in each experiment). In contrast, the Pearson's coefficients of co-localization of GLUT4*myc* with furin or internalized transferrin were only 0.30 and 0.37, respectively. These data suggest that, at steady-state, the majority of GLUT4 is found in a perinuclear sub-compartment that is distinct from sorting/recycling endosomes or the general TGN.

### Internalized GLUT4 sorts into a Syntaxin-6-positive perinuclear sub-compartment and exhibits insulin-responsive re-exocytosis within 30 min

**Fig. 2.**
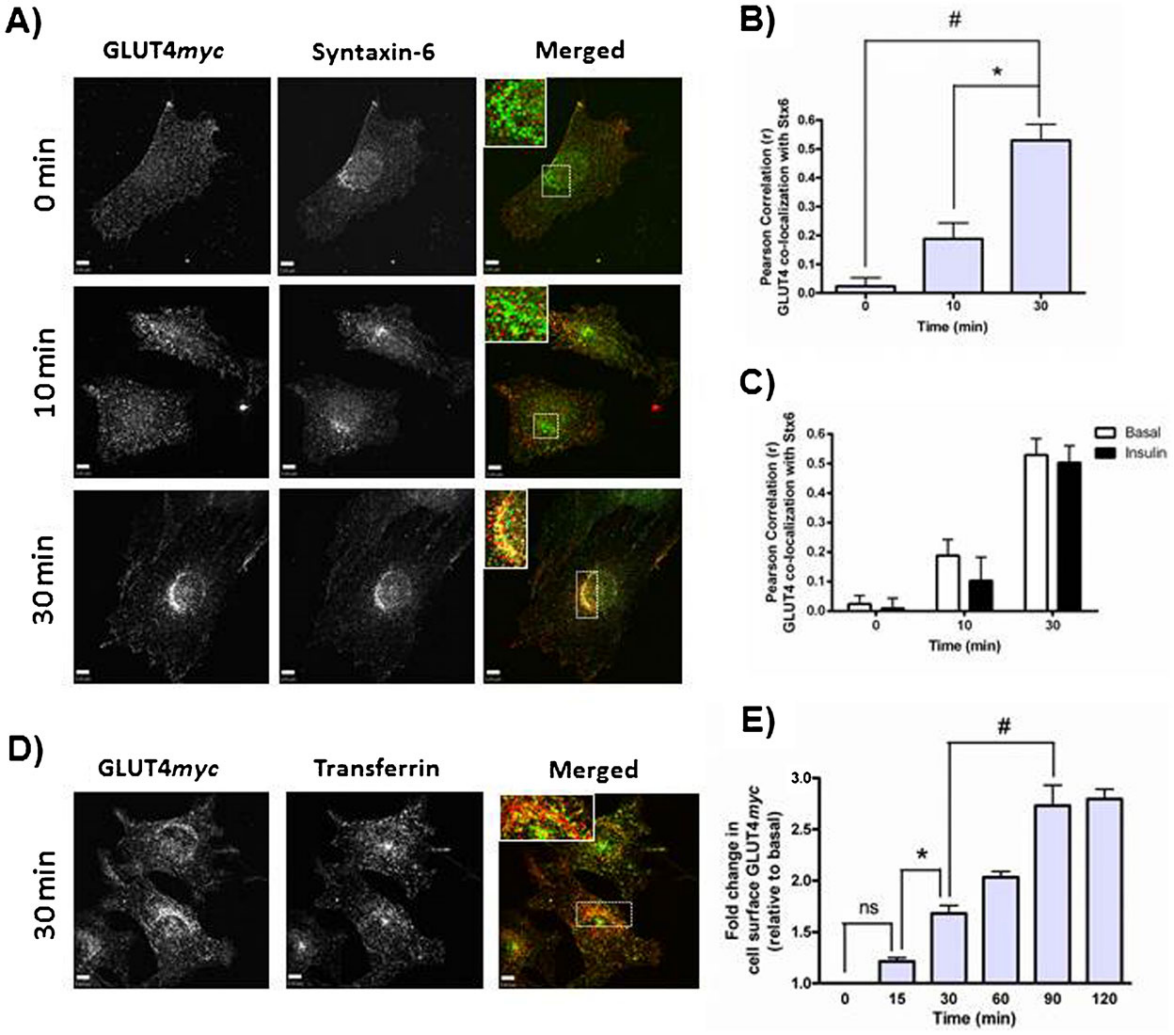
Internalized GLUT4 sorts into a Syntaxin-6-positive perinuclear sub-compartment. (A) Cell surface GLUT4*myc* was labeled at 4°C before cells were re-warmed to 37°C for 0, 10, or 30 min. Fixed cells were then stained for endogenous Stx6 (green). Inset  =  single optical slice of the perinuclear region. (B) Quantification of the co-localization between GLUT4*myc* and Stx6 using Pearson's correlation coefficients (N = 3, 10–15 cells per experiment), *p<0.01; #p<0.001. (C) Internalized GLUT4 sorts into a Syntaxin-6-positive perinuclear sub-compartment independent of insulin. Cells were treated as in panel A, with or without 100 nM insulin for 20 min prior to cell surface labeling of GLUT4*myc*. Insulin was also present during cell re-warm. Pearson correlations were used to quantify the co-localization between GLUT4 and Stx6 (N = 3, 10 cells per experiment). (D) Internalized GLUT4*myc* segregates away from Transferrin. Same protocol as in panel A, but Transferrin Receptor was labeled with A488-conjugated Transferrin for 30 min prior to GLUT4 labeling. Inset  =  single optical slice of the perinuclear region. (E) Internalized GLUT4*myc* undergoes insulin-responsive re-exocytosis by 30 min (N = 2), *p<0.05; #p<0.01. All cells were treated with insulin for 15 min before being placed at 4°C and cell surface GLUT4*myc* labeled. Cells were re-warmed to 37°C for indicated times, treated with insulin for 10 min, and then fixed at 4°C for detection of cell surface GLUT4*myc*. Scale bars: 5 µm.

We next tracked the transit of GLUT4*myc* as it internalizes from the cell surface and proceeds through intracellular compartments. Cell surface GLUT4*myc* was pulse-labeled with anti-*myc* antibody at 4°C, the antibody was then removed, and GLUT4*myc* was allowed to internalize at 37°C. By 10 min, internalized GLUT4*myc* was present in cytosolically dispersed *puncta*, and by 30 min, internalized GLUT4*myc* accumulated in a perinuclear sub-compartment that also contained Stx6 (Fig. 2A, inset). The Pearson's correlation for the co-localization of internalized GLUT4*myc* and Stx6 was statistically significant after 30 min (Fig. 2B). This sub-compartment was devoid of recycling TfnR, as cells pre-labeled with Tfn conjugated to A488 for 30 min prior to GLUT4*myc* labeling showed no overlap between Tfn and internalized GLUT4*myc* at the perinuclear region (Fig. 2D). Some cytosolic puncta were positive for both Tfn and GLUT4*myc*, indicative of GLUT4*myc* in recycling endosomes. This intracellular distribution of internalized GLUT4*myc* is similar to that observed above when total, steady-state GLUT4*myc* was labeled. Insulin did not alter the co-localization between internalized GLUT4*myc* and Stx6 at 10 min or 30 min time points (Fig. 2C). We next tested whether internalized GLUT4*myc* could be recruited back to the plasma membrane in response to insulin (insulin-responsive re-exocytosis). GLUT4*myc* first exhibited insulin-responsive re-exocytosis by 30 min following its internalization (Fig. 2E). GLUT4*myc* that had been allowed to equilibrate for 90 min showed a greater insulin-responsive re-exocytosis, illustrating that a larger population of the surface pulse-labeled GLUT4*myc* had equilibrated with the insulin-responsive compartment. These findings suggest that internalized GLUT4 accumulates in a Stx6-positive perinuclear sub-compartment that may be comprised of, or at least give rise to, insulin-responsive vesicles (GSV/IRV).

### C2-ceramide inhibits insulin-responsive GLUT4 re-exocytosis by disrupting GLUT4 sorting

**Fig. 3.**
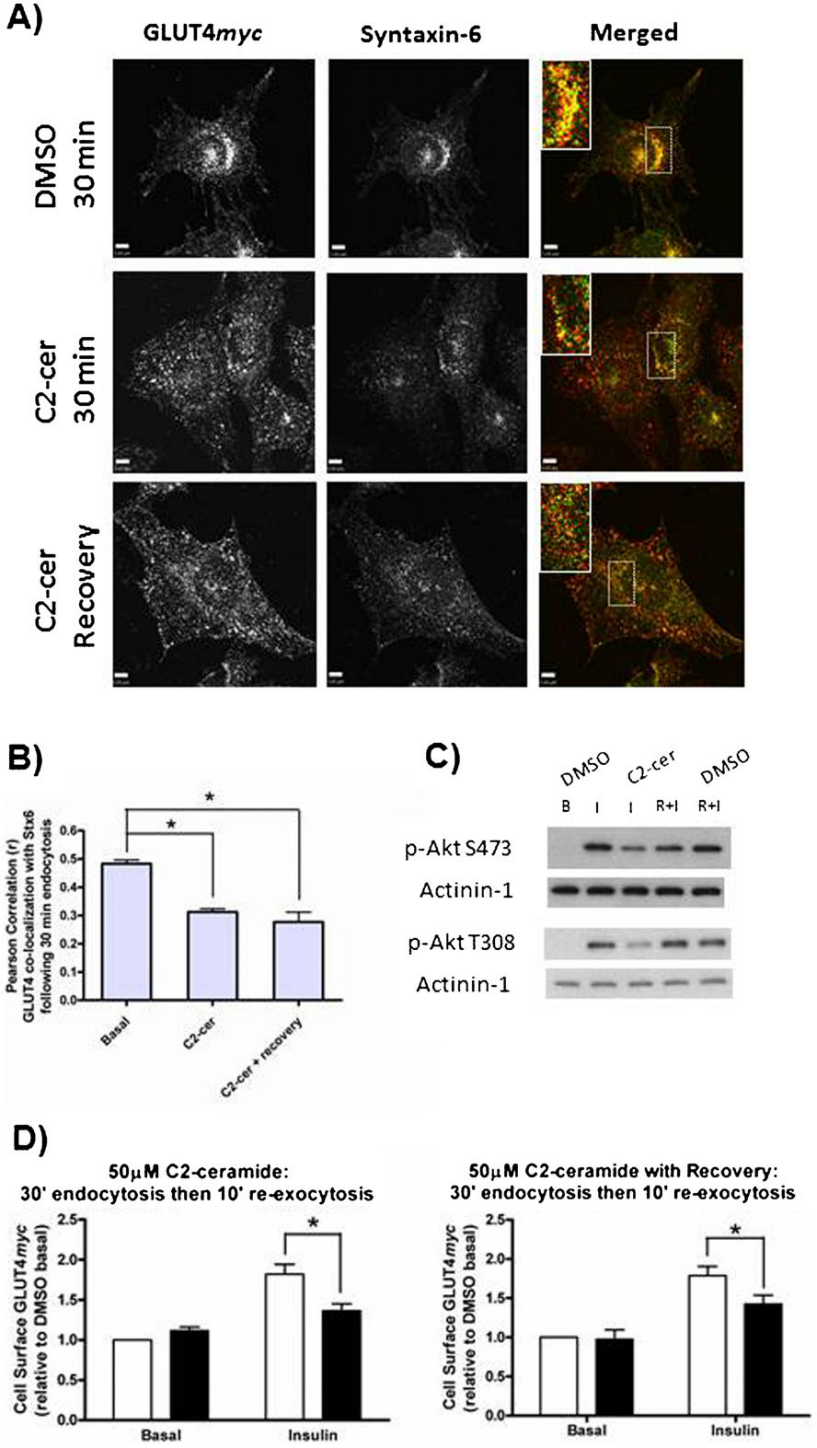
C2-ceramide inhibits both GLUT4 sorting into the Syntaxin-6-positive perinuclear sub-compartment and insulin-responsive GLUT4 re-exocytosis. (A) Cell surface GLUT4*myc* was labeled at 4°C before cells were re-warmed at 37°C for 30 min. C2-ceramide (50 µM) treatments were as described in Materials and Methods. Following re-warm, fixed cells were stained for endogenous Stx6 (green). Inset  =  single optical slice of the perinuclear region. (B) Quantification of the co-localization between GLUT4*myc* and Stx6 using Pearson's Correlation coefficient (N = 3–4, 10–20 cells per experiment), *p<0.01. (C) C2-ceramide inhibits phosphorylation of Akt, but this effect is reversible (N = 2). Cells were treated with C2-ceramide (50 µM) as described for GLUT4 re-exocytosis in Materials and Methods, B  =  basal; I  =  Insulin; R+I  =  recovery + insulin. (D) Insulin-responsive GLUT4 re-exocytosis is inhibited by C2-ceramide. Both 50 µM C2-ceramide (left panel; N = 6, *p<0.001) and 50 µM C2-ceramide with 45 min recovery (right panel; N = 3, *p<0.05) inhibited GLUT4 re-exocytosis. White bars  =  DMSO, Black bars  =  C2-ceramide. Scale bars: 5 µm.

Lipid overload *in vivo* leads to insulin resistance, and this effect has been linked to the accumulation of intracellular ceramides (Chavez and Summers, 2012). Treatment with C2-ceramide is an acknowledged strategy to confer insulin resistance to both L6 muscle cells (JeBailey et al., 2007) and 3T3-L1 adipocytes (Summers et al., 1998). The conferred insulin resistance is so far ascribed to interference with insulin signaling (JeBailey et al., 2007; Powell et al., 2003; Stratford et al., 2001), and the possibility that C2-ceramide also inhibits GLUT4 sorting has been neglected. We treated L6GLUT4*myc* cells with 50 µM C2-ceramide for 2 h prior to pulse-labeling cell surface GLUT4*myc* for subsequent internalization. This treatment dispersed Stx6 out of its perinuclear locale and prevented the perinuclear accumulation of internalized GLUT4*myc* (Fig. 3A, 2^nd^ row, and Fig. 3B). Moreover, the cytosolically dispersed Stx6 and GLUT4*myc* did not colocalize with each other. As expected, treatment with C2-ceramide inhibited insulin-responsive GLUT4*myc* re-exocytosis (by 55%, Fig. 3D, left panel), concomitant with the inhibition of Akt phosphorylation (Fig. 3C, C2-cer I).

Next, we examined whether these effects of C2-ceramide would be reversible and if so, would recovery apply to GLUT4 sorting and its insulin responsiveness. C2-ceramide was first administered for 2 h as above, then washed out for a total of 45 min. Cells were incubated in ceramide-free medium for 15 min prior to pulse-labeling the surface GLUT4*myc* and allowing it to internalize for 30 min (‘45 min washout’). In spite of the C2-ceramide washout, both internalized GLUT4*myc* and Stx6 remained cytosolically dispersed and did not recover co-localization (Fig. 3A, 3^rd^ row, and Fig. 3B). However, this 45 min C2-ceramide washout allowed for the restoration of insulin-dependent Akt phosphorylation upon a subsequent insulin challenge (Fig. 3C, C2-cer R+I). Notably, in spite of the recovery of this crucial step in insulin signaling, the C2-ceramide washout did not restore GLUT4 insulin responsiveness. Insulin-induced GLUT4*myc* re-exocytosis remained inhibited by 46% after the C2-ceramide washout (Fig. 3D, right panel). These results suggest that impairing GLUT4 sorting *per se* may contribute to insulin resistance. They also endorse the concept that failure of GLUT4 sorting to a perinuclearly-located, Stx6-positive sub-compartment causes insulin resistance.

### Microtubule disruption inhibits both GLUT4 sorting into the Syntaxin-6-positive perinuclear sub-compartment and insulin-responsive GLUT4 re-exocytosis

**Fig. 4.**
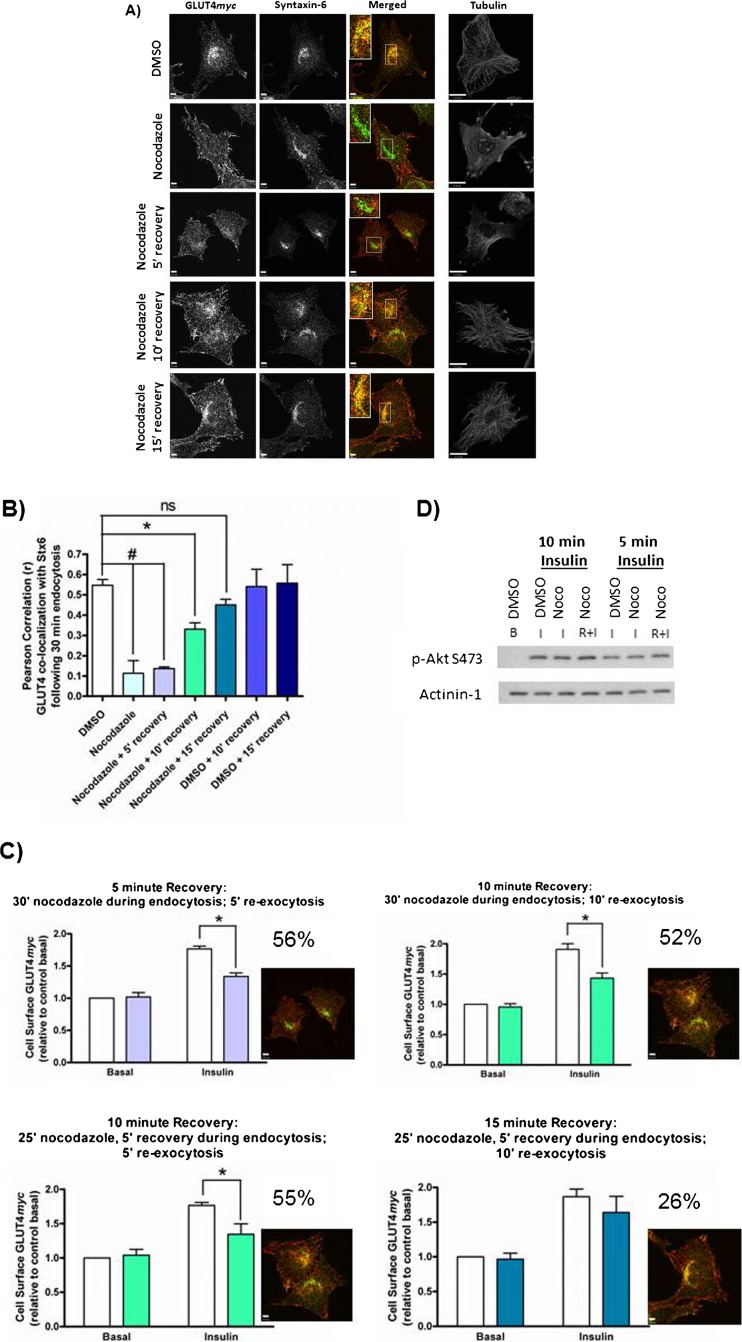
Nocodazole prevents both GLUT4 sorting into the Syntaxin-6-positive perinuclear sub-compartment and insulin-responsive GLUT4 re-exocytosis. (A) Cell surface GLUT4*myc* was labeled at 4°C before cells were re-warmed at 37°C for 30 min with or without 3 µM nocodazole. For recovery, nocodazole was washed out after 25 min and replaced with serum free medium for indicated recovery times. Fixed cells were stained for endogenous Stx6 (green). Inset  =  single optical slice of the perinuclear region. Representative tubulin staining is shown for each condition (far right). (B) Quantification of the co-localization between GLUT4*myc* and Stx6 using Pearson's Correlation coefficient (N = 2–4, 10–15 cells per experiment), *p<0.05; #p<0.001. (C) Western blot showing phosphorylation of Akt at S473 is not affected by 3 µM nocodazole treatment. Noco I  =  nocodazole for 30′ prior to insulin stimulation; Noco R+I  =  nocodazole for 25′ with 5′ recovery prior to insulin stimulation. (D) Nocodazole inhibition of insulin-responsive GLUT4 re-exocytosis is reversible. Cells were treated for re-exocytosis with and without recovery from nocodazole treatment as described in Materials and Methods. Percent inhibition is stated for each condition. Inset shows merged image of GLUT4 (red) co-localization with Stx6 (green) at each time point (from panel A). White bars  =  DMSO, color bars  =  Nocodazole; *p<0.01. Scale bars: 5 µm.

To examine the relationship between Stx6 and GLUT4 sorting, we tested the effect of strategies that would preclude GLUT4 from reaching the perinuclear region and examined its insulin-responsive re-exocytosis. Cell surface pulse-labeled GLUT4*myc* was allowed to internalize for 30 min in the presence of a low dose (3 µM) of nocodazole (Fig. 4A). This treatment disrupted microtubules but did not disrupt the perinuclear localization of Stx6. However, nocodazole effectively prevented the accumulation of internalized GLUT4*myc* in the perinuclear region and accordingly its co-localization with Stx6 (Fig. 4A, 2^nd^ row). In addition to dispersing internalized GLUT4*myc*, nocodazole prevented insulin-dependent GLUT4*myc* re-exocytosis (Fig. 4C, top left panel). Importantly, insulin signaling to Akt was not affected under any of these conditions (Fig. 4D), illustrating an uncoupling between insulin signaling and loss of GLUT4 insulin responsiveness.

Microtubule disruption was reversible upon washing out nocodazole for 5 min (Fig. 4A, 3^rd^ row). By 10 min after nocodazole removal, internalized GLUT4*myc* was still cytosolic and not insulin-responsive (Fig. 4A, 4^th^ row, and Fig. 4C, aqua bars); however, by 15 min internalized GLUT4*myc* re-acquired its perinuclear localization in the Stx6-positive perinuclear sub-compartment (Fig. 4A, 5^th^ row, quantification in Fig. 4B) as well as its insulin-responsive re-exocytosis (Fig. 4C, bottom right). The co-localization of pulse-labeled GLUT4*myc* with Stx6 (from Fig. 4A) is quantified in Fig. 4B (bars color coded to match re-exocytosis in Fig. 4C). Collectively, these results suggest that sorting of internalized GLUT4 to the Stx6-positive perinuclear sub-compartment coincides with its acquisition of insulin-responsiveness.

### Syntaxin-6 is required for insulin-responsive GLUT4 re-exocytosis but not for GLUT4 accumulation in the perinuclear region

**Fig. 5.**
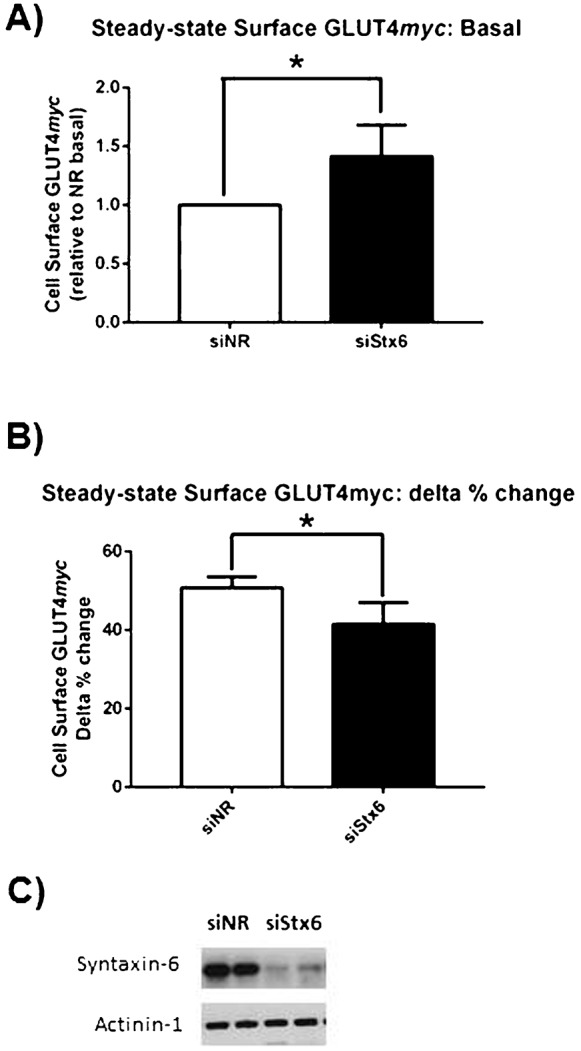
Stx6 depletion increases cell surface GLUT4 and inhibits insulin-stimulated GLUT4 translocation. Cells were treated with or without insulin for 15 min before being fixed for the detection of cell surface GLUT4*myc* (N = 7). (A) Cell surface GLUT4*myc* in unstimulated cells, *p<0.01. (B) Cell surface GLUT4*myc* in insulin-stimulated cells, expressed as delta % change [(Insulin−Basal)×100/Insulin] in cell surface GLUT4*myc* in response to insulin stimulation, *p<0.01. (C) Representative Western blot of Stx6 knockdown.

To test whether Stx6 is required for GLUT4 sorting and insulin responsiveness, the expression of Stx6 was silenced via cognate siRNA. First, the steady-state localization of GLUT4*myc* was probed in cells depleted of Stx6. Stx6 knockdown did not alter the localization of GLUT4*myc* to the Stx16-positive perinuclear region nor did it shift GLUT4*myc* into furin- or Tfn-positive compartments (supplementary material Fig. S1). However, Stx6-depleted cells had 1.5-fold more GLUT4*myc* at the cell surface and showed a partially diminished gain in surface GLUT4 in response to insulin (Fig. 5).

**Fig. 6.**
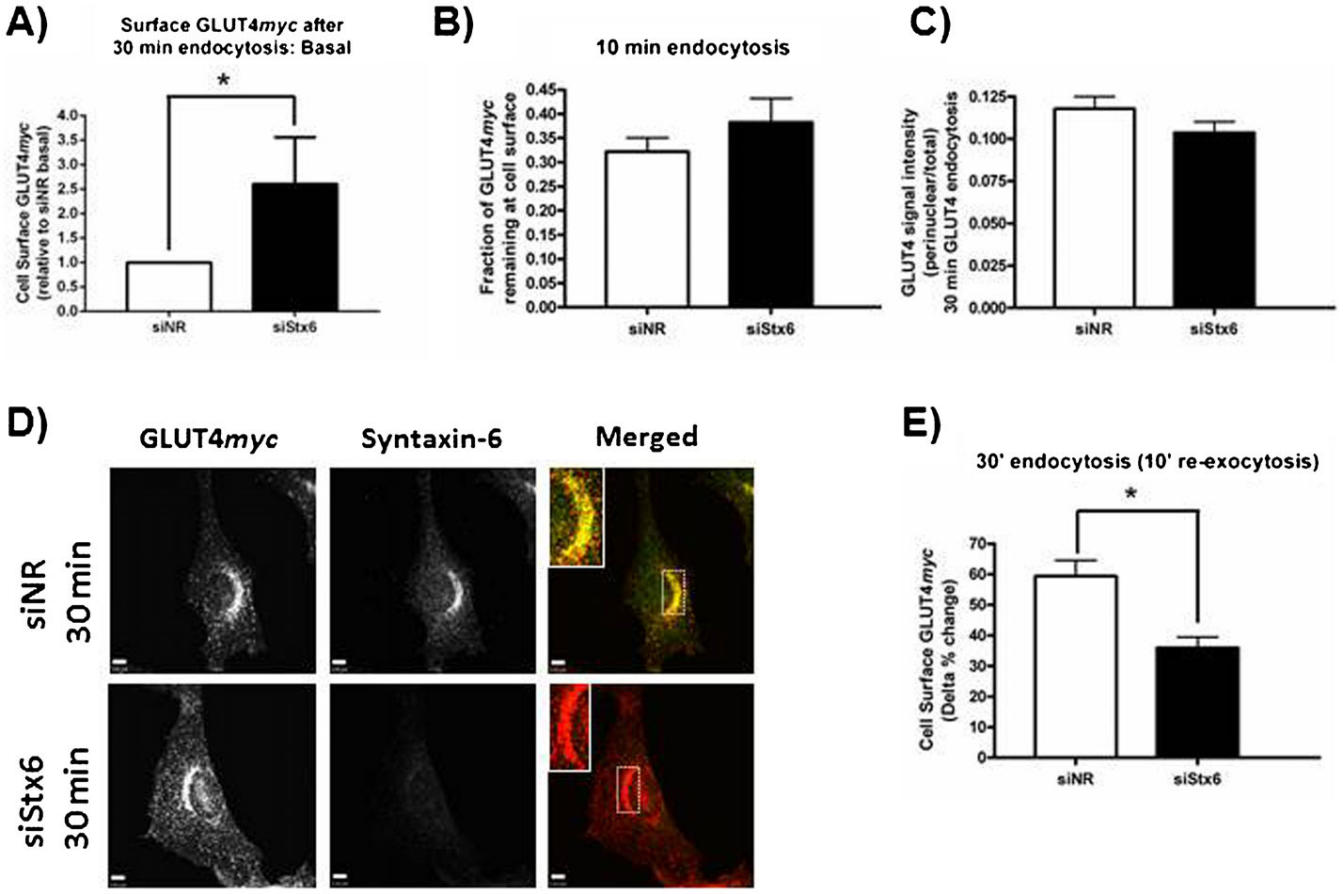
Syntaxin-6 is required for insulin-responsive GLUT4 re-exocytosis but not GLUT4 accumulation in the perinuclear region. (A) Cell surface GLUT4*myc* after 30 min re-warm is higher in cells depleted of Stx6, *p<0.01 (N = 5). All cells were treated with insulin for 15 min before being placed at 4°C and cell surface GLUT4*myc* labeled. Cells were re-warmed at 37°C for 30 min, treated with or without insulin for 10 min, and then fixed at 4°C for detection of cell surface GLUT4*myc*. Cell surface GLUT4*myc* after 30 min re-warm is expressed relative to the siNR control. (B) Stx6 knockdown does not inhibit GLUT4*myc* disappearance from the plasma membrane (N = 6). GLUT4 endocytosis was measured as described in Materials and Methods. GLUT4*myc* remaining at the cell surface after 10 min endocytosis was quantified relative to 0 min. (C,D) Stx6 knockdown does not inhibit GLUT4*myc* accumulation in the perinuclear region. Cell surface GLUT4*myc* was labeled at 4°C before cells were re-warmed at 37°C for 30 min, fixed, permeabilized, and stained for endogenous Stx6 (green). Inset  =  single slice of perinuclear region. GLUT4*myc* accumulation in the perinuclear region is quantified as perinuclear signal intensity over total signal intensity (N = 42 cells over 3 experiments). (E) Stx6 knockdown inhibits insulin-responsive GLUT4*myc* re-exocytosis, *p<0.01 (N = 5). Insulin-responsive re-exocytosis is expressed as the delta % change [(Insulin−Basal)/Insulin] for cells shown in panel A. Scale bars: 5 µm.

The effect of Stx6 knockdown on internalizing GLUT4*myc* was then determined. Stx6-depleted cells also had 2.6-fold more GLUT4*myc* at the plasma membrane than control cells after 30 min internalization (Fig. 6A) and this was not the result of inhibiting GLUT4*myc* removal from the plasma membrane (Fig. 6B). Surprisingly, after 30 min of internalization, GLUT4*myc* still accumulated in a perinuclear region (Fig. 6C,D); however, insulin-responsive GLUT4*myc* re-exocytosis was reduced by 40% (Fig. 6E). Hence, although Stx6 was not necessary for the arrival of internalizing GLUT4 at a perinuclear locale, it was required for GLUT4 dynamic acquisition of insulin-responsiveness. It is likely that the overall perinuclear region contains both constitutively recycling vesicles and the Stx6-positive sub-compartment containing GSV/IRV. Accordingly, Stx6 knockdown may have caused mis-sorting of internalized GLUT4*myc*, leading to its accumulation in perinuclear recycling endosomes that only allow GLUT4 to recycle back to the plasma membrane without proceeding to segregate to acquire insulin responsiveness.

### GLUT4 accumulation in the Syntaxin-6 positive perinuclear sub-compartment correlates with insulin-responsive GLUT4 re-exocytosis

**Fig. 7.**
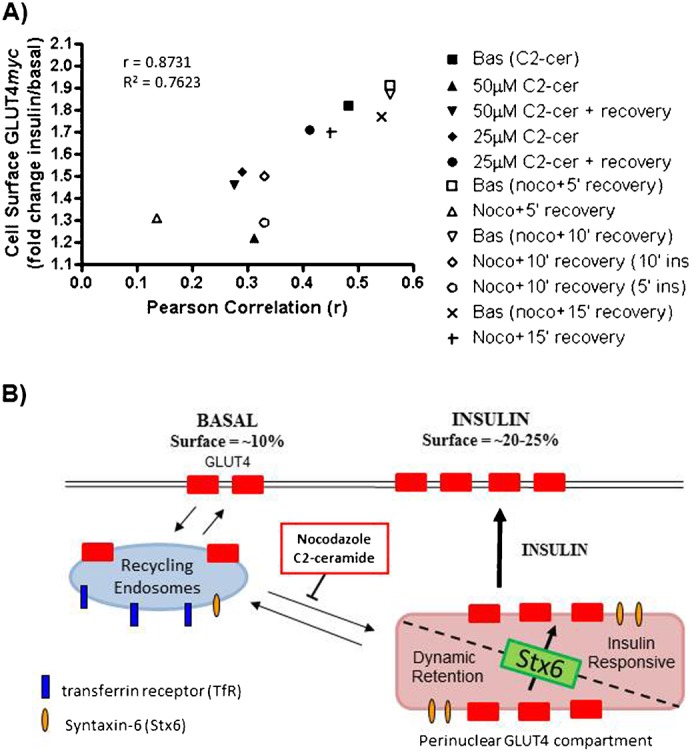
GLUT4 sorting through a Syntaxin-6-positive perinuclear sub-compartment in relation to insulin-responsive exocytosis. (A) GLUT4 co-localization with Stx6 correlates with insulin-responsive GLUT4 re-exocytosis (r = 0.8731, p<0.01). The Pearson Correlation coefficient (*x*-axis) represents GLUT4 co-localization with Stx6 after 30 min of GLUT4 internalization. Cell Surface GLUT4*myc* (*y*-axis) shows the insulin-stimulated change in cell surface GLUT4 re-exocytosis following 30 min of GLUT4 internalization. C2-cer  =  C2-ceramide; Noco  =  nocodazole; C2-cer + recovery  =  ceramide removed for 45 min before GLUT4 re-exocytosis measured; Noco + 5′ recovery  =  25 min nocodazole then 5 min recovery before GLUT4 re-exocytosis measured (+ ins  =  added insulin without nocodazole for stated time). (B) Model of GLUT4 sorting in L6 muscle cells. GLUT4 internalized from the plasma membrane transits recycling endosomes marked by Transferrin Receptor en route to a Stx6-positive perinuclear compartment. This Stx6-positive compartment is comprised of a dynamic retention compartment and insulin-responsive vesicles (GSV/IRV). Stx6 is required to sort GLUT4 from the retention compartment to the insulin-responsive vesicles. Both nocodazole and C2-ceramide prevent GLUT4 from accumulating in the Stx6-positive perinuclear sub-compartment.

To quantify the relationship between GLUT4 accumulation in the Stx6-positive perinuclear sub-compartment and insulin-responsive GLUT4 re-exocytosis, we calculated the correlation of these two parameters across the diverse conditions described in this study. Fig. 7A presents the Pearson's correlation coefficient of the co-localization of Stx6 and internalized GLUT4*myc versus* GLUT4*myc* insulin-responsive re-exocytosis at matched time points. A strong correlation (r = 0.8731) was found between the co-localization of internalized GLUT4*myc* with Stx6 and insulin-responsive GLUT4*myc* re-exocytosis. Furthermore, insulin-responsive GLUT4 re-exocytosis was observed only when Stx6 was expressed and both GLUT4 and Stx6 co-localized in the perinuclear region (Table 1). These data support a model in which GLUT4 internalizing from the plasma membrane is sorted into a perinuclear sub-compartment that is positive for Stx6 but distinct from the recycling endosomes containing recycling TfnR (Fig. 7B). Arrival at this sub-compartment correlates with the acquisition of insulin responsiveness. C2-ceramide inhibits GLUT4 insulin-responsiveness by perturbing the Stx6-positive sub-compartment and GLUT4 sorting to it.

**Table 1.**
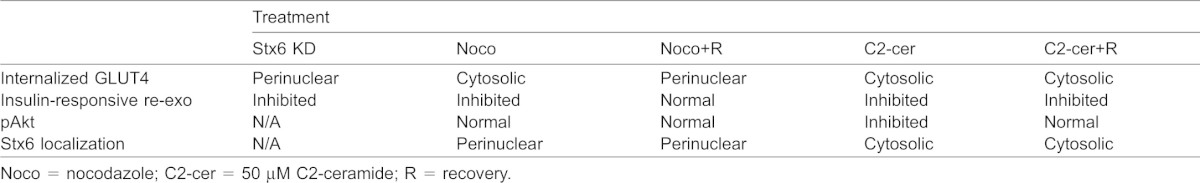
Summary of GLUT4 re-exocytosis parameters measured under each treatment condition

## DISCUSSION

In contrast to the extensive studies on the insulin-dependent signals regulating GLUT4 exocytosis, there is little consensus on the defining mechanisms of GLUT4 sorting into the insulin-responsive compartment (Foley et al., 2011; Stöckli et al., 2011). From this lapse it has become clear that studying the steady-state distribution of GLUT4 as the only endpoint does not allow scrutiny of the intracellular steps required for GLUT4 to attain insulin responsiveness. As an alternative approach, we have followed the internalization of GLUT4 pulse-labeled at the surface of L6 muscle cells to examine its sorting into intracellular compartments and to probe the effects of insulin resistance on this sorting. We provide evidence that GLUT4 internalized from the plasma membrane enters a segregated perinuclear sub-compartment denoted by Stx6, and that insulin resistance induced by C2-ceramide inhibits both this sorting and insulin-responsive GLUT4 re-exocytosis, even when insulin signaling to Akt has been restored. We further show that GLUT4 sorting into the Stx6-positive perinuclear sub-compartment correlates with the acquisition of insulin-responsiveness and that Stx6 participates in GLUT4 sorting into the insulin-responsive compartment. These findings suggest that certain conditions conferring insulin resistance may act by disrupting GLUT4 sorting in the absence of insulin, additionally to or independently from changes in Akt signaling.

### Previous studies on Syntaxin-6 and GLUT4 in adipocytes

To date, the participation of Stx6 in GLUT4 traffic in muscle cells was unexplored, yet physiologically muscle is the major tissue responsible for insulin-dependent glucose disposal, and the cell biology of GLUT4 traffic in muscle and adipose cells shows similarities but also differences (Foley et al., 2011).

In 3T3-L1 adipocytes, GLUT4 co-localizes with Stx6 at the perinuclear region (Li et al., 2009; Shewan et al., 2003). Although several studies have examined the relationship between Stx6 and GLUT4 in adipocytes and shown that internalized GLUT4 accumulates in a Stx6-positive perinuclear compartment (Perera et al., 2003; Shewan et al., 2003; Watson et al., 2008), the functional contribution of Stx6 to GLUT4 sorting and acquisition of insulin responsiveness is still outstanding. Over-expressing the cytosolic domain of Stx6 (cyto-Stx6) – presumably to displace the endogenous Stx6 from its natural location – did not alter insulin-stimulated glucose uptake and instead only elevated basal glucose uptake (Perera et al., 2003). Following insulin removal, cyto-Stx6 delayed GLUT4 internalization from the plasma membrane and prevented GLUT4 recovery in isolated microsomes normally containing GSV (Perera et al., 2003). However, how this related to the acquisition of insulin-responsive exocytosis was not resolved. Hence, more targeted approaches were necessary to study if Stx6 is required for GLUT4 to acquire insulin responsiveness.

While not directly answering that question, Stx6 knockdown in adipocytes inhibited the insulin-stimulated re-exocytosis of Insulin Receptor Amino Peptidase (IRAP) that had been surface-labeled and equilibrated with internal stores for 6 h (Watson et al., 2008). Given that IRAP and GLUT4 co-purify in insulin-responsive microsomes (Kupriyanova et al., 2002), it was suggested that Stx6 may be required for GLUT4 sorting as well. Our findings in muscle cells provide direct evidence that Stx6 is required for GLUT4 sorting into the insulin-responsive compartment and that this compartment is located at the perinuclear region. Moreover, we reveal that insulin resistance conferred by C2-ceramide is due in part to disruption of this compartment and the consequent mis-localization of GLUT4.

### Syntaxin-6 defines GLUT4 sorting into an insulin-responsive compartment in muscle cells

The observation that Stx6 knockdown inhibited insulin-responsive GLUT4 re-exocytosis but not the accumulation of internalized GLUT4 in the perinuclear region suggests that Stx6 functions after internalized GLUT4 reaches this location. Our finding that nocodazole prevented both the co-localization of GLUT4 with perinuclear Stx6 and the GLUT4 response to insulin is consistent with the idea that the two events are functionally linked. Previous studies in 3T3-L1 adipocytes had found that a longer nocodazole treatment for 1–2 h did not inhibit GLUT4 translocation, despite preventing GLUT4 accumulation at the perinuclear region (Molero et al., 2001; Shigematsu et al., 2002). However, in those conditions nocodazole dispersed the entire GLUT4 pool, not only the internalizing fraction, and unfortunately the localization of Stx6 was not investigated. Potentially, nocodazole failed to inhibit GLUT4 translocation because the massive GLUT4 dispersal into the cytosol may have allowed re-formation of functional GLUT4-Stx6 fragmented sub-compartments. In line with this hypothesis, a later study found that the shorter (30 min) treatment with nocodazole did preclude insulin-dependent GLUT4 exocytosis in adipocytes (Karylowski et al., 2004). Here we used the short treatment with nocodazole (30 min) and found it did not disrupt the steady-state perinuclear pool of GLUT4 of muscle cells (data not shown), and, under these conditions the perinuclear localization of Stx6 was also unaffected. Yet, internalizing GLUT4*myc* did not reach this sub-compartment and concomitantly it did not acquire insulin responsiveness. The duration of nocodazole treatment may thus be critical for GLUT4 traffic, but of course it is also plausible that the fine-tuning of GLUT4 sorting by microtubules is somewhat distinct in adipose and muscle cells.

The perinuclear GLUT4/Stx6 compartment is likely devoid of recycling endosomes, in so far as Tfn recycling from the membrane did not reach this compartment. Because GLUT4 is mobilized from the perinuclear location to the cytosol and periphery in response to insulin (Boguslavsky et al., 2012; Fujita et al., 2010; Randhawa et al., 2008), the perinuclear locale is linked to insulin responsiveness. However, we cannot currently distinguish if the perinuclear sub-compartment containing internalized GLUT4*myc* and Stx6 is strictly a storage site (GSV proper) or if it constitutes the bona fide insulin-responding compartment (IRV proper). Ostensibly, GSV proceed to IRV but both functions have been ascribed in the literature to the compartment ambiguously denoted GSV/IRV. The storage function may be where decisions are made to progress to insulin responsiveness or to slowly recycle back to the plasma membrane (Fig. 7B). This scenario is consistent with the finding that Stx6 knockdown increases the amount of GLUT4 at the cell surface. If Stx6 defines GLUT4 progression from GSV to IRV, then in the absence of Stx6, GLUT4 would be prevented from sorting from perinuclear GSV into actual IRV, thus becoming more available for constitutive recycling.

While we find that Stx6 is required for acquisition of insulin-responsiveness by internalized GLUT4, other proteins undoubtedly contribute to the control of GSV and IRV. In addition to Stx6, Stx16 and Vps45 have been implicated in sorting GLUT4 into the GSV/IRV (Perera et al., 2003; Proctor et al., 2006; Roccisana et al., 2013). Although the locus of action is unclear, Stx6 and Stx16 function in a t-SNARE complex at the TGN to capture incoming transport vesicles (reviewed by Johannes and Popoff, 2008). Notably, Vps45 is a Sec1-Munc18 (SM) protein that binds Stx16 and is proposed to regulate the function of the Stx16 SNARE complex involved in sorting GLUT4 into the GSV/IRV (Roccisana et al., 2013). Our results are consistent with a model where Stx6 may function as part of the t-SNARE complex that regulates GLUT4 sorting from constitutively recycling endosomes into the GSV/IRV or from GSV into IRV (Fig. 7). This model suggests that Stx6 functions on GSV/IRV membranes, possibly with Stx16 and Vps45, to capture GLUT4-containing vesicles into the insulin responsive compartment. Other events contributing to GLUT4 sorting into GSV/IRV include luminal interactions with sortilin to recruit GGA adaptors (Li and Kandror, 2005; Shi and Kandror, 2007) and input from the small G proteins Rab11 and Rab14 (Reed et al., 2013; Sadacca et al., 2013; Zeigerer et al., 2002). Interestingly, recent studies position Rab14 at the exit from recycling endosomes into a GLUT4 retention compartment (Reed et al., 2013; Sadacca et al., 2013).

### Ceramide-induced insulin resistance involves an insulin signal-independent defect in GLUT4 sorting

Insulin resistance can arise from defects in insulin signaling events (typically at the levels of IRS-1 or Akt) (Foley et al., 2011; JeBailey et al., 2007; Summers et al., 1998; Xiong et al., 2010). Obesity and lipid overload are associated with elevated intramuscular and circulating levels of ceramides that contribute to insulin resistance (Chavez and Summers, 2012). While an underlying mechanism is inhibition of Akt activation (Stratford et al., 2001), effects alternative to inhibition of signaling had not been considered. Here, we have provided evidence that C2-ceramide inhibits GLUT4 sorting into the Stx6-positive perinuclear sub-compartment and that this occurs even when insulin stimulation of Akt is allowed to recover. We further show that defects in GLUT4 sorting alone, such as those imposed by nocodazole or Stx6 knockdown, can inhibit an ensuing insulin-responsive GLUT4 exocytosis event.

### Conclusions

The mechanism whereby GLUT4 is sorted into insulin-responsive vesicles is poorly understood. Current models, developed mostly in adipocytes, rely largely on the study of steady-state GLUT4 localization to infer modes of GLUT4 sorting. Here we report that, in muscle cells, GLUT4 internalized from the plasma membrane sorts into a Stx6-positive, perinuclear sub-compartment devoid of furin or Tfn, and provide evidence that GLUT4 sorting through this sub-compartment is a prerequisite for acquisition of insulin responsiveness. Furthermore, we show that C2-ceramide inhibits GLUT4 sorting into this compartment thereby possibly underlying the observed inhibition of insulin-responsive GLUT4 exocytosis. Our data are consistent with a model where ceramide could cause insulin resistance by altering intracellular GLUT4 sorting.

## Supplementary Material

Supplementary Material
