# Supplementary material for: Dynamic GLUT4 sorting through a syntaxin-6 compartment in muscle cells is derailed by insulin resistance-causing ceramide
Source: Biol Open. 2014 Apr 4;3(5):314–25. doi: 10.1242/bio.20147898 (PMC4021353; doi:10.1242/bio.20147898)
Supplement: Supplementary Material [file supp_3_5_314__index.html]

Dynamic GLUT4 sorting through a syntaxin-6 compartment in muscle cells is derailed by insulin resistance-causing ceramide — Dynamic GLUT4 sorting through a syntaxin-6 compartment in muscle cells is derailed by insulin resistance-causing ceramide — Supplementary Material 

# Dynamic GLUT4 sorting through a syntaxin-6 compartment in muscle cells is derailed by insulin resistance-causing ceramide

## bio.20147898 Supplementary Material

**Files in this Data Supplement:**

- Supplementary Material - Kevin P. Foley and Amira Klip doi: 10.1242/bio.20147898
