# Supplementary material for: Dynamic GLUT4 sorting through a syntaxin-6 compartment in muscle cells is derailed by insulin resistance-causing ceramide
Source: Biol Open. 2014 Apr 4;3(5):314–25. doi: 10.1242/bio.20147898 (PMC4021353; doi:10.1242/bio.20147898)
Supplement: Supplementary Material [file supp_bio.20147898_bio.20147898-s1.pdf]

Biology Open (2014) 000, 1–12 doi:10.1242/bio.20147898

Supplementary Material

Kevin P. Foley and Amira Klip doi: 10.1242/bio.20147898

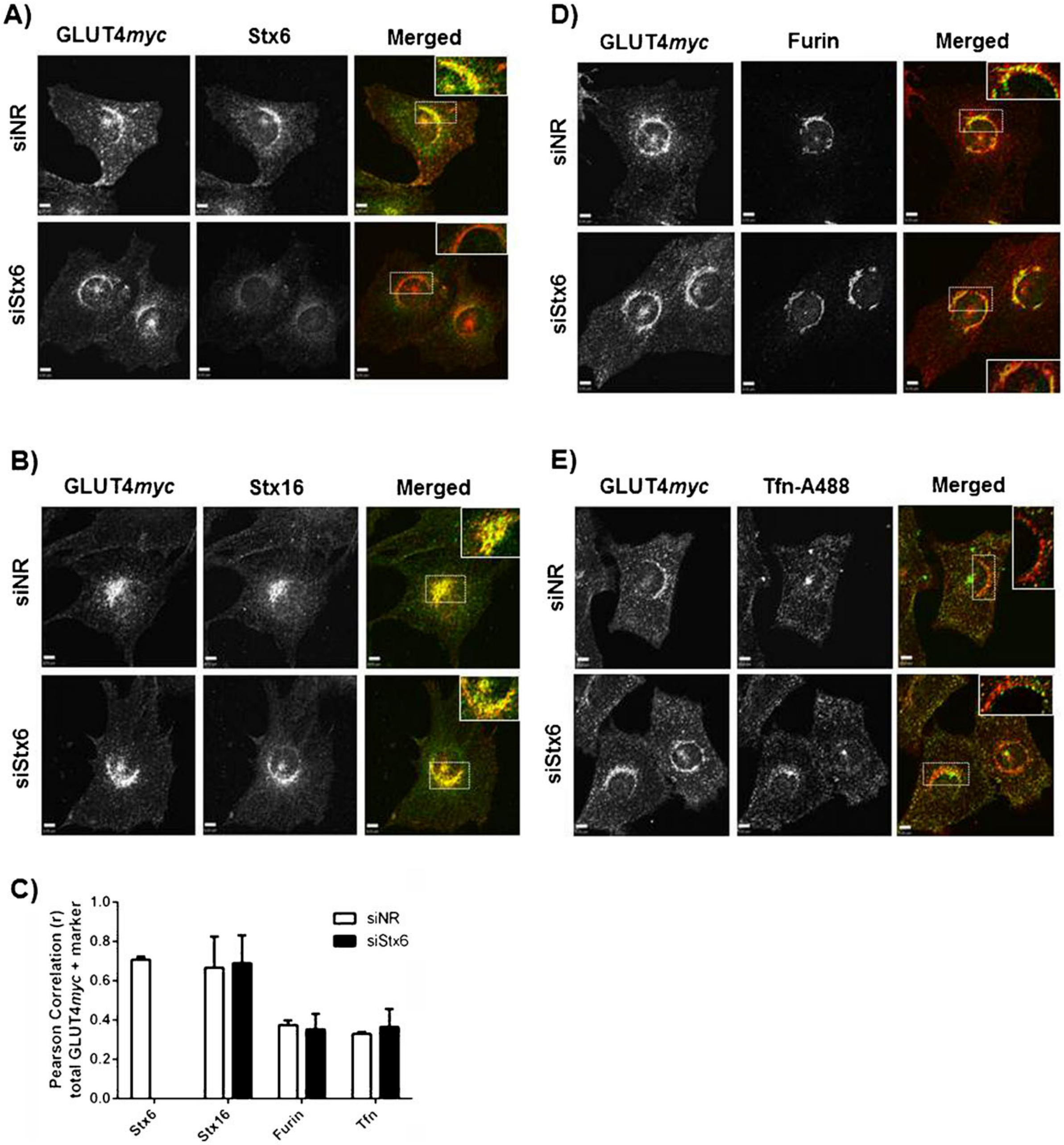

Fig. S1. Stx6 depletion does not alter the steady-state localization of GLUT4. Cells were fixed, permeabilized, and labeled for GLUT4*myc* (red) and Stx6 (green) (A) or GLUT4*myc* (red) and Stx16 (green) (B). Inset = single optical slices of the perinuclear regions. (C) GLUT4*myc* co-localization with markers of endomembrane compartments was quantified using Pearson correlations. Single cells were selected and Pearson correlations calculated using Volocity software (N=2). 8–15 cells were quantified in each experiment. (D) Cells were fixed, permeabilized, and labeled for GLUT4*myc* (red) and furin (green). Inset = single slice of perinuclear region. (E) Cells were loaded with Tfn-A488 for 30 min before being fixed, permeabilized, and labeled for GLUT4*myc* (red). Inset = single optical slice of the perinuclear region. Scale bars: 6 µm.
